# Intralipid emulsion therapy for ivermectin toxicity in a dog

**DOI:** 10.1002/vms3.70586

**Published:** 2025-08-26

**Authors:** Ehsan Khaksar, Hamed Karimi, Mahdieh Rezaei, Salar Zehforosh

**Affiliations:** ^1^ Department of Veterinary Clinical Science G.C Islamic Azad University Garmsar Iran; ^2^ Department of Clinical Science, Faculty of Veterinary Medicine Shahid Bahonar University of Kerman Kerman Iran

**Keywords:** dog, intralipid, ivermectin, toxicity

## Abstract

**Objective:**

Therapeutic management of ivermectin overdose in a Shih Tzu dog using intravenous administration of intravenous lipid emulsion (ILE).

**Case Summary:**

A 3‐year‐old female Shih Tzu dog weighing 2.5 kg was presented to a veterinary clinic in Tehran with ataxia, tremors, incoordination, negative PLR and menace reflex, bradycardia and salivation. The patient was treated with a 10 mg ivermectin tablet with the brand name Medimect in the last 12 h. Following the diagnosis of ivermectin intoxication, treatment with ILE was initiated. ILE was administered as a bolus of 1.5 mL/kg in 1 min, followed by a dose of 0.25 mL/kg/min for 30 min as a CRI. The neurological symptoms of the patient began to decrease 2.5 h after the ILE therapy. Twenty‐four hours later, the patient was resuscitated, and all symptoms were resolved.

**Conclusion:**

The current paper reports the successful treatment of ivermectin toxicity with ILE therapy in Iran. The education of veterinarians regarding the beneficial and varied roles of ILE therapy in different clinical settings is warranted, particularly in terms of the potential for ILE therapy to reverse the toxicities of various lipophilic drugs.

## Graphical abstract

Ivermectin is used to treat external parasites such as demodex by binding to glutamate and GABA receptors, causing parasite paralysis. The intralipid emulsion is widely used in poisoning with lipophilic drugs such as ivermectin, and its ease of use in emergency cases has made it applicable. Though the mechanism of action is not understood, it is postulated that ILE provides a lipid compartment within circulating plasma in which the toxic compounds become sequestered, known as the ‘lipid sink’ phenomenon.

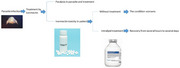

## 1 Introduction

Intravenous lipid emulsion (ILE) has emerged as a potent antidote for the treatment of drug poisoning in the past (Ozcan and Weinberg 2014). ILE therapy has been introduced as a promising treatment for systemic poisoning caused by local anaesthetics (Bates et al. 2013; Weinberg and Riou 2012). Moreover, recent reports have shown that it is also considered in other types of drug poisoning (Ozcan and Weinberg 2014). Currently, ILE is widely used in poisoning with lipophilic drugs such as ivermectisn, and its ease of use in emergency cases has made it applicable (Cave and Harvey 2009). Though the mechanism of action is not understood, it is postulated that ILE therapy provides a lipid compartment within circulating plasma in which the toxic compounds become sequestered, known as the ‘lipid sink’ phenomenon (Fernandez et al. 2011). In blood, the fat droplets of ILE form a lipid compartment, separate from the plasma aqueous phase, into which a lipophilic substance might dissolve. Thus, a lipophilic drug might be drawn into this ‘lipid sink’, reducing the lipophilic drug aqueous plasma concentration (Weinberg et al. 1998). Based on this lipid sink theory, the lipophilicity (characterised by log *P*) of the toxin is highly correlated with the efficacy of ILE therapy in decreasing toxic effects (Peacock et al. 2015).

Ivermectin is used as a macrocyclic lactone to treat external parasites such as Demodex and Sarcoptes mites and internal parasites of nematodes in dogs. By binding to glutamate and GABA receptors, it causes parasite paralysis (Campbell 1989). In mammals, the administration of this drug has a good safety margin in cases of a healthy blood–brain barrier (BBB) ​​and also the use of the appropriate drug dose (Merola and Eubig 2018). Mutations in the ATP‐binding cassette sub‐family B member 1 (ABCB1) gene in some dogs, as well as insufficient BBB development in immature puppies, may result in ivermectin crossing the BBB and the development of neurological symptoms (Mealey 2004). Alternatively, high concentrations of ivermectin can cross the BBB without the aid of the gene mutation and still lead to toxicity in dogs (Martin 1997). The ivermectin toxicity is usually associated with neurological symptoms including ataxia, blindness, mydriasis, tremors, disorientation and depression to coma (Jourdan et al. 2015). The current article is the report of the successful management of ivermectin toxicity in a Shih Tzu dog in Iran using ILE therapy.

## 2 Patient History and Clinical Observations

A 3‐year‐old female Shih Tzu dog weighing 2.5 kg had been referred to a veterinary clinic in Tehran. In the clinical examination, various symptoms were observed, including neurological signs of ataxia, tremors, incoordination and depression; ophthalmologic signs of negative PLR and menace and dazzle reflexes and bilateral mydriasis; gastrointestinal sign of drooling; respiratory sign of hypoventilation; and cardiovascular sign of bradycardia. Ten minutes after entering the clinic, the patient became laterally recumbent on the triage table. It had been found in the history that 12 h before referring to the clinic, this dog was referred to another clinic because of a skin disease. There, after skin scraping, the diagnosis of demodicosis was confirmed, and she was treated with 10 mg ivermectin tablets with the brand name Medimect. According to the history and clinical examination, the diagnosis of ivermectin toxicity was confirmed for this patient, and then therapeutic management was done. Blood samples were taken from the patient for haematology and biochemical tests prior to and 24 h after treatment (Table 1).

**TABLE 1:** Haematology and biochemical tests before and 24 h after treatment.

Parameter (unit)	Reference interval	Before ILE	After ILE
HTC (%)	37–55	46	42
WBC (× 10^3^/mcL)	6.0–17.0	11.6	10.8
Platelets (× 10^3^/mcL)	148–484	256	240
Total protein (g/L)	54–71	66	60
Albumin (g/L)	26–33	30	27
Globulin (g/L)	27–44	36	33
Glucose (mg/dL)	65–118	99	105
Triglyceride (mg/dL)	40–169	84	149
Total bilirubin (mg/dL)	0–0.4	0.2	0.3
BUN (mg/dL)	10–28	30	22
Scr (mg/dL)	0.5–1.7	1.1	0.9
ALP (U/L)	33–150	115	195
ALT (U/L)	16–40	32	34

## 3 Treatment

A bolus of 50 mL of normal saline was administered intravenously to the patient. Then, 1.5 mL/kg (3.75 mL) of ILE 20% (SMOFLIPID 20%) was injected intravenously as a bolus, followed by 0.25 mL/kg/min (18.75 mL) as CRI for 30 min in accordance with previous recommended protocols (Markert et al. 2023; Allerton 2020). Improved tremors and slow return of PLR were observed 2.5 h following the initial administration of ILE. After 12 h, the patient fully regained menace and PLR reflexes. Neurological symptoms such as ataxia, incoordination and tremors were completely resolved after 24 h (Table 2).

**TABLE 2:** Clinical symptoms observed in the poisoned patient.

Clinical signs	Observed items
Neurologic signs	Ataxia, tremor, incoordination, depression
Cardiovascular signs	Bradycardia
Ophthalmologic signs	Negative PLR and menace and dazzle reflex
Gastrointestinal signs	Salivation/drooling
Respiratory signs	Hypoventilation
Evidence of haemorrhage	
Allergic reactions	

In CBC, and biochemical tests before and after treatment, the only significant abnormality was the increase of triglycerides and ALP. The increase of triglycerides was not enough to lead to hypertriglyceridemia and its related problems (its increase was within the normal range). The increase in ALP after treatment could be due to direct damage to the bile ducts, but this increase was not severe enough to lead to cholestasis. The increase in BUN in the first test can be attributed to prerenal azotaemia or the patient's lack of fasting.

## 4 Discussion

Ivermectin, as one of the most widely used drugs in veterinary medicine, may cause neurotoxicity in high doses (Epstein and Hollingsworth 2013). Based on various sources of veterinary pharmacology, the recommended dose for treating demodicosis with ivermectin is 0.1–0.6 mg/kg; however, the dose should be increased to 0.6 mg/kg slowly with regard to the side effect tolerance (Plumb 2018). The primary mechanism of action of ivermectin is enhancing glutamate and GABA receptors (Gallagher et al. 2008). Following the binding of ivermectin to the receptors, the chloride channels open slowly and irreversibly, resulting in long‐term hyperpolarisation or depolarisation of the neurones or myocytes, which results in rapid paralysis. In the case of injecting a high dose of ivermectin, the drug is absorbed much faster, but the symptoms of poisoning can be observed in all methods of administration with a higher dose (Simon et al. 2019). In the past, several reports have described the poisoning caused by the administration of ivermectin and its treatment with ILE. During a study conducted in 2023 by Carina and her colleagues, it was found that ILE was also effective in the treatment of poisoning with permethrin, moxidectin, ibuprofen and lidocaine (Markert et al. 2023). In this case, ivermectin was prescribed at a toxic dose of 2.2 mg/kg (3.5 times the standard dose), which led to severe neurologic signs, but the overdose poisoning was completely resolved in this report by administering the standard dose of ILE) without any side effects over 24 h.

Although ILE has previously been used for parenteral nutrition, its use as an antidote to treat toxicity caused by lipophilic substances is an additional application for this drug (Ozcan and Weinberg 2014; Bartlett 2014). This drug is used in human medicine in the treatment of poisoning caused by lipophilic local anaesthetics and cardiotoxic drugs and can lead to a significant improvement in the clinical condition (Bates et al. 2013).

ILEs may contain varying types (soybean, coconut, olive and fish) and concentrations of triglyceride oils (10%, 20% and 30%). Droplets have similar sizes and shapes to physiological chylomicrons and are similarly metabolised (Jaffal et al. 2023). There are different ILEs with different trade names that have different percentages of soybean oil, medium‐chain triglycerides, olive oil, fish oil, egg lipid and glycerine in the pharmacopoeias of countries. Some of the most famous trade names are INTRALIPID20%, SMOFLIPID20% and LIPOVENOES10%. Intralipid is the most often studied product among ILEs. It is an emulsion containing 20% soybean oil, 2.25% glycerol and 1.2% egg phospholipids (Buys et al. 2015). In this patient, we used SMOFLIPID20% to neutralise the toxicity caused by ivermectin. SMOFLIPID20% contains soybean oil (60 g/L), medium‐chain triglycerides (60 g/L), olive oil (50 g/L), fish oil (30 g/L), egg phospholipids (12 g/L), glycerol (25 g/L) and vitamin E (200 mg/L). Based on past studies and reference articles, the dose of ILE 20% for the treatment of poisoning with some lipophilic drugs, including ivermectin, is 1.5–5 mL/kg intravenously as a bolus, followed by 0.25–0.50 mL/kg/min as CRI for 30–60 min (Markert et al. 2023; Allerton 2020).

Overall, the mechanism of action of ILE is not well understood, but there are many theories. The two most widely accepted theories are the ‘lipid sink’ theory and the ‘improved myocardial performance’ theory (Jaffal et al. 2023). The ‘lipid sink’ or ‘PK sequestration’ theory is the most researched one. Triglyceride oils in ILE exhibit a high affinity for lipophilic drugs and make a lipid‐rich PK compartment within the bloodstream, drawing them away from their targets and free blood. A new equilibrium is established thereafter, reducing lipophilic drug tissue concentrations and helping organs work better again, allowing the drug's metabolism and elimination. ILE inactivates a portion of lipophilic drugs by acting as alternative binding surfaces so they cannot reach their target organs (Weinberg et al. 1998). The cardiotonic effects can be explained by the combined scavenging effect and a direct cardiac effect, which clarify why recovery from a drug overdose occurs rapidly (Fettiplace et al. 2015). ILE is able to exert direct physiological effects on the heart and vessel, improving cardiac output (Fettiplace et al. 2013), which may facilitate the accelerated redistribution of the toxicant. The underlying mechanisms allowing ILE‐attributed improvement in cardiac output have not been fully explored. The infused volume of ILE is a major contributor but not the only one, and other factor are still unknown. The calcium influx and fatty acid hypothesis is the most well‐known. Fatty acids are supposed to increase calcium influx in the myocardial cells to make a positive inotropic effect (Ozcan and Weinberg 2014; Gueret et al. 2007).

The effectiveness of ILE has been improved with supportive treatments such as fluid therapy (Jaffal et al. 2023). The improvement of clinical symptoms in this type of poisoning may be slowed with ILE therapy. The poisoned animal should be closely monitored for at least a week since more treatments may be needed (Simon et al. 2019).

One of the side effects of using ILE is hypertriglyceridemia, which has been reported in both humans (Levine et al. 2014; Cave et al. 2014) and animals (Hiller et al. 2010). In humans, severe hypertriglyceridemia often leads to pancreatitis (Patel et al. 2014). Hypertriglyceridemia can cause intravascular lipid deposition in the liver, brain, kidneys and other organs. Lipid overload syndrome can explain fever, hepatomegaly, splenomegaly (or rarely pancreatitis), anaemia, leukopenia, thrombocytopenia, haemolysis, coagulopathy and hepatic dysfunction in animals (Hojsak and Kolaček 2014). Seldom, corneal lipidosis has been described in cats (Yuh and Keir 2018), but it has not been reported in dogs. In this report, the patient had no evidence of hypertriglyceridemia 24 hours following ILE therapy and complete recovery. Reports illustrate that hyperlipemia syndrome caused by ILE therapy may depend on both the types of animal species and individual differences, as well as the amount of drug injected (Yuh and Keir 2018). In most of the reports that ILE therapy led to hypertriglyceridemia, CRI was performed for more than 1 h; however, in our patient, CRI was administered for only 30 min, which may explain the absence of lipemic serum.

In addition to the clinical manifestations, hypertriglyceridemia may interfere with the results of several routine biochemical tests. The degree of interference depends on the specific assay used by the laboratory, the species (canine vs feline), as well as the severity of the hypertriglyceridemia. In addition, hyperlipidaemia may cause haemolysis, which in turn can interfere with the results of some biochemical assays. Occasionally, hyperbilirubinemia may cause the cholesterol concentration to be falsely lowered. These potential alterations in biochemical data must be considered when results of testing in animals with hyperlipidaemia are interpreted. Fortunately, many laboratories will endeavour to clear the hypertriglyceridemia by ultracentrifugation before performing biochemical assays (Jacobs et al. 1992; Nelson and Couto 2019).

Other adverse effects are possible in cases of multiple doses or long‐term use (Jaffal et al. 2023). During ILE therapy as CRI, the patient should be carefully monitored for febrile and allergic reactions, and if these symptoms are observed, CRI should be stopped, especially during the first 20 min (Robben and Dijkman 2017). Prolonged treatment may be required depending on the dose of ivermectin administered/ingested by the patient and the MDR1 mutation status of the patient. Prognosis is usually very good with aggressive supportive care administered in the early stages. Clinical signs may take several weeks to resolve (Simon et al. 2019). If the dose is greater than 5 mg/kg in dogs without MDR1 mutation, prognosis may be guarded (Merola et al. 2009).

The severity of poisoning with macrocyclic lactones, even at low doses, can be increased due to a mutation in the canine gene ABCB1 (previously known as MDR1), which leads to the production of a defective p‐glycoprotein in the vascular endothelial cells that form the BBB (Londoño et al. 2017). In cases of severe intoxication, patients may experience significant hypoventilation or respiratory paralysis, necessitating mechanical ventilation. However, with timely and appropriate treatment, the prognosis can be quite favourable (Hopper et al. 2002).

In a study by Geraldine in 2015, 20 cats that were poisoned by ivermectin were treated with ILE therapy. It was noticed that many of the cats started to get better within the first day after receiving an initial dose of ILE and continuing treatment. The other cats recovered after being treated with ILE in a dosage spread across the second and third days (Jourdan et al. 2015). This retrospective case study observed that repeated/prolonged treatment was required if ILE therapy was not effective on the first day. In addition, multiple published case reports of dogs with ivermectin toxicity reported complete recovery when administered ILE bolus and CRI on the first day (Epstein and Hollingsworth 2013; Simon et al. 2019). However, in dogs with the MDR1 gene mutation, clinical signs may be prolonged, and several days of ILE therapy may be necessary for complete recovery (Clarke et al. 2011).

In this case, the patient recovered within 24 h without complications, likely due to immediate presentation after clinical signs began and prompt treatment.

## 5 Conclusion

The current paper is the first report of successful treatment of ivermectin poisoning with ILE therapy in Iran. In view of these observations and previous comparable work, further work may be conducted to obtain named ILE as an antidote to ivermectin in the near future.

## Author Contributions

**Ehsan Khaksar**: Methodology, project administration, supervision, resources. **Hamed Karimi**: Writing – original draft, methodology, writing – review and editing, data curation, software, project administration. **Mahdieh Rezaei**: Investigation, validation, writing – review and editing, formal analysis, supervision. **Salar Zehforosh**: Writing – original draft, methodology, writing – review and editing, data curation, supervision, resources.

## Ethics Statement

The authors have nothing to report.

## Conflicts of Interest

The authors declare no conflicts of interest.

## Peer Review

The peer review history for this article is available at https://www.webofscience.com/api/gateway/wos/peer‐review/10.1002/vms3.70586.

## Data Availability

All data generated or analysed during this study are included in this submitted article (and its supplementary information files).
